# Therapy of Type 2 Diabetes in Patients with SARS-CoV-2 Infection

**DOI:** 10.3390/ijms22147605

**Published:** 2021-07-16

**Authors:** Weronika Bielka, Agnieszka Przezak, Andrzej Pawlik

**Affiliations:** Department of Physiology, Pomeranian Medical University in Szczecin, 70-111 Szczecin, Poland; weronika.bielka@wp.pl (W.B.); agn-prze@wp.pl (A.P.)

**Keywords:** COVID-19, diabetes, therapy

## Abstract

COVID-19 infection poses an important clinical therapeutic problem, especially in patients with coexistent diseases such as type 2 diabetes. Potential pathogenetic links between COVID-19 and diabetes include inflammation, effects on glucose homeostasis, haemoglobin deoxygenation, altered immune status and activation of the renin-angiotensin-aldosterone system (RAAS). Moreover, drugs often used in the clinical care of diabetes (dipeptidyl peptidase 4 inhibitors, glucagon-like peptide 1 receptor agonists, sodium-glucose cotransporter 2 inhibitors, metformin and insulin) may influence the course of SARS-CoV-2 infection, so it is very important to verify their effectiveness and safety. This review summarises the new advances in diabetes therapy and COVID-19 and provides clinical recommendations that are essential for medical doctors and for patients suffering from type 2 diabetes.

## 1. Introduction

Coronavirus disease 2019 (COVID-19), caused by infection with severe acute respiratory syndrome coronavirus 2 (SARS-CoV-2), currently affects many people worldwide. Studies have found an increased severity of COVID-19 or a high risk of death in patients with advanced age, male sex, cardiovascular disease, obesity and type 1 (T1DM) or type 2 diabetes mellitus (T2DM) [1]. Both T2DM and COVID-19 infection are associated with ethnicity, socioeconomic status and metabolic factors such as metabolic syndrome [2]. Furthermore, COVID-19 infection might also create a predisposition to hyperglycaemia in people without impaired glucose tolerance [3]. The main entry receptor for SARS-CoV-2 in human cells is angiotensin-converting enzyme 2 (ACE2), which is highly expressed in lung alveolar cells, vascular endothelium, cardiac myocytes and many other cell types [4], although dipeptidyl peptidase 4 (DPP4) might also act as a binding target [5]. SARS-CoV-2 infection not only induces mild symptoms, but also has the potential to develop into systemic inflammatory response syndrome, acute respiratory distress syndrome (ARDS), multi-organ involvement and shock [6]. Potential pathogenetic links between COVID-19 and diabetes include inflammation, effects on glucose homeostasis, haemoglobin deoxygenation, altered immune status and activation of the renin-angiotensin-aldosterone system (RAAS) [3]. Moreover, drugs that are often used in the clinical care of type 2 diabetes (dipeptidyl peptidase 4 inhibitors, glucagon-like peptide 1 receptor agonists, sodium-glucose cotransporter 2 inhibitors, metformin and insulin) may influence the course of SARS-CoV-2 infection, so it is very important to verify their effectiveness and safety.

## 2. Potential Pathophysiological Mechanisms Existing in Diabetes and COVID-19

### 2.1. Effects on Glucose Homeostasis

The presence of hyperglycaemia or typical complications of diabetes increases COVID-19 severity and mortality [1]. An association exists between hyperglycaemia and dysregulation of the innate and humoral immune system. Hyperglycaemia may cause impaired phagocytosis and bactericidal activity, neutrophil chemotaxis, complement fixation and opsonisation of microorganisms, as well as altered chemokine production [7]. Moreover, elevated glucose concentrations and glycolysis support SARS-CoV-2 replication in human monocytes via the activation of hypoxia-inducible factor 1α (HIF-1α) and the production of mitochondrial reactive oxygen species [8]. Endothelial damage by inflammation, glucotoxicity, oxidative stress and cytokine production leads to an increased risk of thromboembolic complications and the risk of damage to vital organs in patients with diabetes [3]. A typical complication of COVID-19 in patients with diabetes is glycaemic deterioration, which causes the rapidly increasing need for high doses of insulin in patients requiring insulin treatment [9]. In patients with a severe course of COVID-19, viral-induced inflammation may affect the functions of the skeletal muscle and liver, which are the major organs responsible for insulin-mediated glucose uptake, thereby increasing insulin resistance [10]. Furthermore, ketoacidosis frequently occurs in patients with COVID-19, either T1DM or T2DM [11]. A study of 174 participants has demonstrated that patients with diabetic ketoacidosis and infectious shock were more likely to die of the virus [12].

### 2.2. Inflammation

In patients with COVID-19, IL-6, TNF-α and IFN-γ have been confirmed to be critical pathogenic cytokines involved in the inflammatory storm [13]. Blood levels of IL-6 and lactate dehydrogenase (LDH) are independent predictors of COVID-19 severity [14]. A retrospective study of 317 patients with laboratory-confirmed COVID-19 showed a correlation between these indicators of active inflammatory response within 24 h of hospitalisation and disease severity [14]. Moreover, blood levels of IL-6 might correlate with a pro-coagulant profile [15]. IL-6 may cause damage to lipids, proteins and DNA by increasing oxidative stress, which, as a consequence, leads to an impairment of the body’s structure and function and causes the rapid progression of COVID-19 in patients with diabetes [3]. The presence of other inflammatory markers, such as D-dimers and ferritin, might contribute to an increased risk of microvascular and macrovascular complications in patients with underlying diabetes [16]. In a French study, these complications were significantly associated with an increased risk of mortality in patients with diabetes and COVID-19 comorbidity [17]. On the other hand, in a Chinese study, patients with COVID-19 and elevated glucose levels showed promoted cytokine profiles and immune responses [18]. In that study, patients with coexisting diabetes had shorter hospitalisation times [18]. The possible explanation for this phenomenon is that increased blood glucose levels might be beneficial for some anti-inflammatory cytokines and for the process of eliminating the virus [18].

Respiratory syncytial virus infections increase IFN-γ production, which, as a result, activates natural killer (NK) cells as a defensive mechanism and also causes insulin resistance in human muscle and adipose tissues [19]. A connection exists between NK cell activity and glycaemic deterioration in patients with impaired glucose metabolism [20]. NK cell activity is lower in patients with T2DM than in those with prediabetes or normal glucose tolerance [20]. Reduced NK cell activity might be one explanation for why patients with diabetes are more susceptible to COVID-19 and have a worse prognoses [3].

### 2.3. Activation of the RAAS

In a healthy human, an equilibrium exists between the action of angiotensin II and angiotensin (1–7). Angiotensin II is made by angiotensin-converting enzyme, ACE, from angiotensin I and has vasoconstrictory and pro-thrombotic properties [21]. On the other hand, ACE2 can hydrolyse angiotensin II to angiotensin (1–7), which causes vasodilatation and acts in an antithrombotic manner through the production of prostacyclin and nitric oxide [22,23]. Moreover, angiotensin II determines the overproduction of IL-6, TNF-α and other pro-inflammatory cytokines [24]. It has been suggested that the prevalence of angiotensin II during SARS-CoV-2 infection may lead to the exacerbation of the cytokine storm and cause acute respiratory distress syndrome and multi-organ dysfunction [21]. Furthermore, ACE2 may serve as an entry receptor for SARS-CoV-2 and is expressed in many human cells, including pancreatic islets [25,26]. Hyperglycaemia might lead to the induction of aberrant glycosylation of the ACE2 receptor, which promotes the binding of SARS-CoV-2 to the ACE2 receptor [27]. On the other hand, COVID-19 infection is able to cause hyperglycaemia in people without pre-existing diabetes [28]. This finding and the localisation of ACE2 expression in the endocrine pancreatic cells suggests that SARS-CoV-2 specifically damages pancreatic islets, potentially leading to hyperglycaemia [28]. There is a possibility that the damage of pancreatic beta cells and increased insulin resistance, as well as impaired glucose metabolism, caused by COVID-19 may lead to the occurrence of diabetes in the future [29].

### 2.4. Haemoglobin Deoxygenation

Glycosylated haemoglobin is a deoxygenated form of haemoglobin and its level is higher in diabetic patients than in people without hyperglycaemia. Deoxyhaemoglobin may be more easily attacked by the surface proteins of SARS-CoV-2. This observation suggests that there is an increased risk of COVID-19 infection for diabetic patients. The proteins bind to the 1-β chain of the haem in the haemoglobin of erythrocytes. As a consequence, iron is dissociated to form porphyrin. This phenomenon causes a loss of function of red blood cells in oxygen and carbon dioxide carrying, leading to respiratory distress symptoms [30].

### 2.5. Altered Immune Status

**Figure 1:**
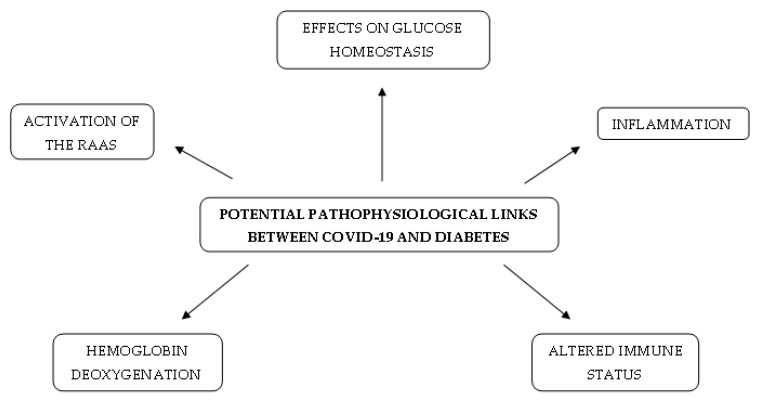
Potential pathophysiological links between COVID-19 and diabetes.

The accumulation of HIF-1α in the hyperglycaemic state leads to the upregulation of LDH activity [31]. In addition, in severe SARS-CoV-2 infection, LDH levels are frequently higher than usual [32]. Lactate may play an important role in the modulation of the inflammatory immune response. It inhibits the retinoic acid-inducible gene I-like receptor (RLR) by binding directly to the mitochondrial antiviral-signalling protein transmembrane domain. This, as a consequence, decreases IFN production and viral clearance [33]. In the aforementioned mechanism, increased lactate production in diabetes may attenuate RLR signalling and delay the clearance of SARS-CoV-2, causing severe outcomes in patients with COVID-19 and diabetes [34]. A short summary of potential pathophysiological mechanisms existing in diabetes and COVID-19 is presented in Figure 1.

## 3. Diabetes Treatment and COVID-19

### 3.1. Dipeptidyl Peptidase 4 Inhibitors in COVID-19

The aforementioned potential pathophysiological mechanisms existing in diabetes and COVID-19 are of crucial importance in the context of diabetes treatment during SARS-CoV-2 infection. Taking into consideration all types of antidiabetic drugs, dipeptidyl peptidase 4 (DPP4) inhibitors (DPP4is), gliptins, are our first focus of attention regarding their impact on the course of COVID-19.

Dipeptidyl peptidase 4 is an aminopeptidase which is expressed on the surface of various cell types. It also circulates in the blood as a soluble form (sDPP4), maintaining its activity as an enzyme there [35]. This peptidase contributes mainly to the regulation of glucose metabolism and the degradation of incretins—hormones that lead to lower blood glucose concentrations through, among other mechanisms, increasing insulin secretion after oral food intake [36]. Furthermore, DPP4 plays a major role in the immune system. Not only is it a marker of activated T lymphocytes, but it also cleaves many peptide hormones, chemokines and proteins which show immunomodulatory properties [35]. In addition, DPP4 possibly participates in the modification of acquired and innate immune responses [37,38]. The binding of adenosine deaminase to dipeptidyl peptidase 4 plays a vital role in providing co-stimulatory signals to T lymphocytes, by increasing the degradation of adenosine, a strong suppressor of T cells, and thus promoting the activation and proliferation of T cells [39]. Moreover, adenosine deaminase has been shown to block the binding of MERS-CoV to cell-bound DPP4 and thus protect it from entering into host cells [40]. Analogous properties of this deaminase have not been confirmed in COVID-19, but cannot be excluded. DPP4 also modulates the function of other immune cells and also stimulates the production of pro-inflammatory cytokines [41].

DPP4 is known to be the main entry receptor for the Middle East Respiratory Syndrome coronavirus (MERS-CoV) [42], but in silico modelling has shown that SARS-CoV-2 probably interacts with this enzyme as well as with ACE2 [5,43]. This could be an explanation for why the spread of SARS-CoV-2 infection in various tissues runs so easily and why the virus shows tropism to either the respiratory or gastrointestinal epithelium. Increased levels of DPP4 might promote the development of SARS-CoV-2 infections in patients with coexisting diabetes and obesity and may be responsible for the more severe course of the disease in this population because of the support of subclinical chronic inflammation and immune system dysregulation [44].

The soluble form of DPP4 might enhance the responsiveness of memory cells to antigens and stimulate the proliferative response of T lymphocytes, possibly through interfering with its form located in membranes and, eventually, protecting T cells from anergy or apoptosis [45]. It has been found that the level of plasma sDPP4 is lower in patients suffering from MERS and correlates with the severity of disease [46], suggesting a protective role for the soluble enzyme. A similar conclusion about decreased sDPP4 levels has been reached regarding patients with the severe course of COVID-19 [47]. A possible explanation for this advantageous feature of sDPP4 is that it may react with virus proteins, preventing their interactions with cell-bound DPP4 and thus making viruses unable to enter into targeted tissues.

Gliptins are commonly used antidiabetic medications, which are well tolerable and body-weight-neutral, and have a low risk of hypoglycaemia, thus showing a safe profile [48]. They are inhibitors of DPP4, and not only have an impact on lowering the glucose concentration in blood in patients with diabetes, but also influence other DPP4 properties, including immune effects. It is suggested that using DPP4is may offer some advantages, preventing coronaviruses from entering host cells [49,50] and eliciting an anti-inflammatory effect, as DPP4 is also involved in the development of inflammation [51]. This last claim is supported by some research which has demonstrated a beneficial role of sitagliptin in reducing the plasma concentrations of pro-inflammatory markers [52,53]. The direct effect of gliptins on DPP4, which could prevent MERS-CoV and SARS-CoV-2 from entering into cells, seems to be unlikely, as in silico modelling has shown that binding sites for viral proteins and gliptins are different [43,54]. However, an indirect effect of DPP4i treatment on the reduced invasion of SARS-CoV-2 into targeted cells may exist, which consists of modulating the interaction between dipeptidyl peptidase and caveolin-1, a protein that is essential for the formation of endosomes and entering cells [55]. In vitro studies have shown that treatment with sitagliptin, vildagliptin or saxagliptin did not prevent coronaviruses from entering into cells [42], but the protective role of teneligliptin regarding a mechanism associated with caveolin-1 has been confirmed in a rodent model [56]. So far, there are no clear data which could prove these assumptions regarding possible interactions between DPP4is and SARS-CoV-2 entry into host cells.

Regarding the beneficial role of dipeptidyl peptidase 4 in proper immune system functions, the question is raised whether the inhibition of DPP4 through DPP4is does not deteriorate the immune response and eventually increase the susceptibility to SARS-CoV-2 infection. A meta-analysis studying the overall risk of infections of DPP4is has not confirmed any association between DPP4i treatment and an increased risk of infections in comparison to placebo or another antidiabetic treatment [57]. Large preclinical studies of rodent models have confirmed that the selective inhibition of DPP4 does not impair T-dependent immune responses to antigenic challenges [58]. Furthermore, short periods of DPP4i treatment, as well as long-term use, did not unfavourably affect the lymphocytes and plasma levels of the main cytokines [59,60].

The role of DPP4 inhibitors in the course of COVID-19 remains unknown and the current knowledge clearly supports neither a favourable nor an adverse effect of gliptins during the infection, mainly because of the small groups of patients included in the research and treated with DPP4is, the lack of some data for all patients and the different outcomes taken into consideration. In an Italian multicentre retrospective study of 338 hospitalised patients with COVID-19 and coexisting type 2 diabetes—the “Sitagliptin in Type 2 Diabetes and COVID-19 (SIDIACO)” study—treatment with sitagliptin added to the standard of care was associated with a reduction in mortality, an improvement in clinical outcomes and an increase in hospital discharges [61]. A similar conclusion about the association between DPP4i treatment and reduced mortality was made in a case series study from an academic hospital in Italy [62]. Another retrospective study including 904 diabetic patients with a moderate–severe course of SARS-CoV-2 infection showed that treatment consisting of gliptins had no significant influence on mortality and clinical outcomes [63]. Similar conclusions regarding the lack of a significant influence of DPP4is on the course of COVID-19 came from a retrospective epidemiological study including 85 hospitalised patients with type 2 diabetes [64] and from the large-scale French “Coronavirus SARS-CoV-2 and Diabetes Outcomes (CORONADO)” study) including 1317 diabetic patients [65]. In contrast, in a retrospective, observational cohort study of 717 hospitalised patients, treatment with DPP4is was associated with worse outcomes in diabetic patients, increasing the risk of intensive care unit admission [66]. In the latest meta-analysis it has been reported that DPP4 inhibitor treatment during hospitalisation is associated with lower mortality, but there is no similar correlation when infected patients used DPPis prior to admission [67]. Further research is required to define an exact role of DPPis in the course of COVID-19 in people with coexisting diabetes.

### 3.2. Glucagon-Like Peptide 1 Receptor Agonists in COVID-19

Glucagon-like peptide 1 (GLP1) receptor agonists (GLP1RAs) belong to another type of antidiabetic medications which affect the incretin axis.

Glucagon-like peptide 1 is a protein secreted from the intestinal L cells after oral glucose intake and immediately degraded by dipeptidyl peptidase 4. It exerts its actions through the glucagon-like peptide 1 receptor (GLP1R), expressed in various tissues—the pancreas, kidney, heart, central nervous system, gastrointestinal tract, lung, muscle or adipose tissue [68]. A reduction in the plasma levels of inflammatory markers, such as interleukin 6, intracellular adhesion molecule 1 and biomarkers of oxidative stress, has been reported during the infusion of native GLP1 in patients suffering from type 2 diabetes [69], which indicates a possible beneficial effect of incretins during inflammation.

Glucagon-like peptide 1 receptor agonists seem to have systemic anti-inflammatory properties, which might potentially play a supportive role during SARS-CoV-2 infection. They may interfere with nuclear factor-kappa B (NF-ĸB) signalling pathways [70] and inhibit the release of cytokines, attenuating pulmonary inflammation [71,72]. Moreover, treatments based on GLP1 have been confirmed to reduce not only the production of inflammatory cytokines, but also the infiltration of immune cells in organs such as the liver, kidney and lungs [36,73,74]. Treatment based on these medications shows a reduction in the production of inflammatory cytokines in the respiratory epithelium in a murine model infected with respiratory syncytial virus [75]. Furthermore, in animal studies regarding sepsis, it has been confirmed that the administration of liraglutide improved survival, vascular dysfunction and inflammation, as well as haemostatic indicators [76]. Some research has highlighted a potential positive effect of GLP1RAs in chronic inflammatory diseases, for instance, neurodegenerative disorders [77] or non-alcoholic fatty liver disease [78], probably through a reduction of inflammatory pathway activity [73]. However, it remains unknown whether such effects exerted on low-grade inflammation translate into anti-inflammatory properties during infection with SARS-CoV-2, and further research should be conducted.

The fact that liraglutide has been reported to probably increase the expression of ACE2 in the heart and lungs raised doubts about the safe long-term use of GLP1RAs in patients with diabetes under the conditions of the COVID-19 pandemic as it could increase susceptibility to infection [79,80]. However, studies have not proved this initial belief. In addition, ACE2 has been showed to have a protective role in acute respiratory distress syndrome (ARDS) in lung diseases, partly because of restoring the production of angiotensin (1–7) [81], and the under-expression of ACE2 has been confirmed to be an indicator of infection progression [80,82]. It has also been suggested that the course of SARS-CoV infection may be favourably modulated by ACE2 [83]. Moreover, in animal studies, the overexpression of ACE2 caused by the administration of liraglutide has been associated with negative effects on inflammatory and fibrotic processes [84]. Liraglutide treatment may also stimulate the synthesis of the surfactant proteins A and B by type II pneumocytes [84,85]. SARS-CoV-2 has a confirmed ability to damage type II pneumocytes, leading to a loss of surfactant and alveolar collapse [21]. It is suggested that the ability of glucagon-like peptide 1 receptor agonists to increase the expression of ACE2, and consequently to stimulate the production of surfactant, may protect the type II pneumocytes and prevent ARDS [86].

Because of the advantageous effects of GLP1RA treatment, such as a reduction in cardiovascular events [73], the prevention of cardiovascular disease and kidney disease [87,88], lowering body mass, as well as a reduction of the risk of hypoglycaemia and glucose variability in the setting of the intensive care unit [89], these drugs seem to be a good choice for patients who are at risk of a severe course of COVID-19. However, the initiation of such therapies is not recommended during acute or critical conditions because of the fact that GLP1RAs show a delayed onset of action and might cause nausea or vomiting at the beginning of treatment [90]. Presently, there are no clinical-epidemiological studies which directly indicate a beneficial effect of GLP1RA treatment on the course of COVID-19.

### 3.3. Sodium-Glucose Cotransporter 2 Inhibitors in COVID-19

Sodium-glucose cotransporter 2 (SGLT2) inhibitors (SGLT2is), or gliflozins, are oral antidiabetic drugs which mainly act on the kidneys and inhibit renal glucose reuptake in proximal renal tubes, inducing glucosuria and a decrease in glycaemia.

SGLT2is have been shown to increase the expression of ACE2 in the kidney, similar to GLP1RAs, so an assumption that they may increase susceptibility to infection has been made [79]. On the other hand, the upregulation of ACE2 leads to an increase in the production of angiotensin (1–7), a vasodilator showing anti-oxidative as well as anti-fibrotic properties, and preventing the development of ARDS [91]. These drugs have also been reported to reduce cardiovascular and renal complications, as well as to show anti-inflammatory properties in animal models [92]. In some studies, treatment based on SGLT2 inhibitors in T2DM patients decreased the mRNA expression levels of tumour necrosis factor, IL-6 or monocyte chemoattractant protein 1 [93,94]. Moreover, one of the SGLT2is, dapagliflozin, has been reported to potentially decrease lactic acidosis through various mechanisms and influence the acid–base balance inside a cell under conditions of hypoxia, which could eventually prevent cell injury during the course of COVID-19 [95]. Furthermore, dapagliflozin, by lowering the cytosolic pH and decreasing the viral load, may protect against the severe course of SARS-CoV-2 infection [95].

A large-scale analysis has shown that patients who suffer from cardiometabolic or renal diseases have susceptibility to worse outcomes of COVID-19 [96]. Other pleiotropic effects of gliflozins, such as cardioprotective and nephroprotective properties [97], mean that the attention of scientists and clinicians is turned to SGLT2is as a treatment which could potentially prevent the adverse course of the infection. Currently, no clinical research has investigated an advantageous effect of treatment based on sodium-glucose cotransporter 2 inhibitors in COVID-19. A series from Italy has reported the lack of an influence of empagliflozin on clinical outcomes in severe pneumonia caused by SARS-CoV-2 in three nondiabetic patients [98]. Another retrospective analysis has shown that prior treatment with gliflozins in diabetic patients is associated with a lower risk of mechanical ventilation [66]. The benefits of SGLT2i treatment during COVID-19 remain unknown and are currently being investigated in the Dapagliflozin in Respiratory Failure in Patients with COVID-19 (DARE-19) trial [99].

However, SGLT2 inhibitors may cause euglycaemic diabetic ketoacidosis (DKA), especially in patients with severe acute disorder, dehydration or acute kidney injury [100], and are therefore not recommended for diabetic patients with a moderate or severe course of COVID-19 [101]. In addition, SARS-CoV-2 infection may cause acidosis, ketoacidosis and diabetic ketoacidosis for patients with diabetes [102]. The possible risk of DKA during gliflozin treatment should not be ignored, and some caution is necessary. The results of the aforementioned DARE-19 trial will provide a better understanding of the clinical consequences of the use of sodium-glucose cotransporter 2 inhibitors during COVID-19.

### 3.4. Metformin in COVID-19

Metformin is one of the most commonly used antidiabetic drugs; because of its widespread use, the impact of metformin therapy on the course of COVID-19 is particularly important.

It has been confirmed that metformin has some anti-inflammatory [103] and anti-thrombotic [104] properties, which may prevent the development of cytokine storms or thromboembolic events. This last claim is supported by research, in which metformin treatment was associated with a reduced risk of deep-vein thrombosis in type 2 diabetes [105], or by studies which have proved the ability of metformin to protect platelets against activation and to prevent extracellular mitochondrial DNA from being released [106]. Furthermore, this molecule shows protective properties towards the endothelium, reducing oxidative stress and inflammation [107]. Moreover, it is suggested that metformin might stop the virus from entering target cells by activating AMPK or via the PI3K/Akt signalling pathway [108].

Metformin use has been reported to decrease mortality in T2DM patients with COVID-19 [17,109,110,111]. In the CORONADO trial, regarding all available antidiabetic medications, only metformin treatment showed a reduced rate of death [17]. In a multicentre retrospective study, it has been shown that metformin treatment prior to admission to hospital was correlated with a decreased intensive care unit admission rate in a dose-dependent fashion [112]. In contrast, in a retrospective study, it was indicated that metformin therapy is associated with an increased severity of COVID-19 infection and with a higher number of life-threatening complications [113]; however, in another systematic review, it was shown that metformin might improve the clinical outcomes in diabetic patients with a mild to severe course of COVID-19 [114]. Treatment with metformin has also been proven to promote acidosis, but not mortality, in diabetic patients infected with SARS-CoV-2 [115] and it was initially suggested to avoid metformin treatment in patients suffering from COVID-19 with coexisting DM [101,116,117]. However, researchers have since pointed out the potential benefits of this method for diabetes management [118]. Of note, metformin is still contraindicated in patients with a high risk of acidosis [119] or acute respiratory distress syndrome [116] and watchfulness is required during the continuation of therapy. Unclear evidence should be investigated in further trials to clarify the exact role of metformin in COVID-19.

### 3.5. Thiazolidinediones in COVID-19

Pioglitazone, an example of thiazolidinediones, is an agonist of a nuclear peroxisome proliferator-activated receptor-γ (PPARγ), which is involved in the regulation of the transcription of genes related to glucose and lipid metabolism [120]. It has shown anti-inflammatory activity [121]. Pioglitazone may reduce the secretion of some proinflammatory cytokines in leukocytes, such as monocytes or macrophages [122], as well as it exhibiting the property of blocking caspase recruitment domain-containing protein 9 in macrophages, thus weakening cytokine storms [123]. Moreover, bioinformatic analysis carried out by Wu et al. has shown that pioglitazone probably inhibits RNA synthesis and the replication of SARS-CoV-2 by affecting 3-chymotrypsin-like protease [124]. However, therapy based on pioglitazone was associated with weight gain, fluid retention, oedema and aggravation of heart failure, so it is not recommended for critically ill patients, including patients with COVID-19 [125,126].

### 3.6. Insulin in COVID-19

Insulin treatment has been suggested as a preferable method of diabetes management during the COVID-19 pandemic for critically ill diabetic patients with the infection [71]. Insulin has been reported to downregulate ACE2 receptors in a diabetic mouse model [127], which may hypothetically reduce the risk of infection of SARS-CoV-2. Furthermore, treatment based on intravenous insulin infusion, leading to optimal glycaemia control in patients with T2DM and COVID-19, seems to have a positive effect on inflammation and coagulation, and may be an effective method for achieving specified glycaemic targets [128]. Of note, patients suffering from SARS-CoV-2 infection require significantly higher insulin doses [101], which may be explained by the dysfunction of beta-cells or the high inflammatory process induced by the virus.

It has been reported that patients with COVID-19 who use insulin have a higher risk of poor prognosis than noninsulin users [63]. Moreover, insulin treatment in hospitalised patients has been linked to increased invasive ventilation [112]. However, it should be noted that these groups might have been incomparable, as insulin is usually the only recommended treatment in the severe course of infections and because in an advanced stage of diabetes with some comorbidities other glucose-lowering medications are contraindicated [63]. On the other hand, in the aforementioned CORONADO study, therapy based on insulin was not associated with death [17]. Treatment with subcutaneous insulin has also shown favourable results in uncomplicated diabetic ketoacidosis during the COVID-19 pandemic [129]. Further research is needed to define the exact role of insulin in the course of COVID-19.

A summary of antidiabetic drugs, their mechanisms of action and their possible influence on the course of COVID-19 is presented in Table 1.

## 4. Recommendations on Diabetes Treatment during the Pandemic

It has been proven that poor glycaemic control has a negative impact on prognosis and on the risk of any infections for both uninfected and infected people; thus, the strict management of diabetes is vital [130]. Although there is a lot of consideration about hypothetically increased susceptibility to COVID-19 during particular antidiabetic treatments in uninfected patients, no evidence confirming these assumptions is available, so treatment based on the usual glucose-lowering medications taken previously is recommended [117,131]. It is of crucial importance to intensify the metabolic control of diabetes by uninfected people as a method of the primary prevention of COVID-19 [101]. According to the guidelines of the American Diabetes Association for the year 2021, HbA1c <7% (53 mmol/mol) without significant hypoglycaemia is recommended. For patients with limited life expectancy or in a situation when the harms of treatment are greater than the benefits, HbA1c <8% (64 mmol/mol) may be appropriate. The use of continuous glucose monitoring devices should be also considered in patients with type 2 diabetes with multiple daily injections and other forms of insulin therapy, especially in those with a high risk of hypoglycaemia. Because an increased time in range is associated with the risk of microvascular complications, a goal is a time in range >70% (70–180 mg/dL, 3.9–10.0 mmol/L) with time below target <4% (<70 mg/dL, <3.9 mmol/L). Patients with cardiovascular disease and type 2 diabetes benefit from glucose-lowering therapies with an SGLT2 inhibitor or a GLP-1 receptor agonist. Moreover, in the current situation, people with diabetes should be a priority population for vaccinations. Routine vaccinations for SARS-CoV-2 infection and influenza prevent morbidity and reduce hospitalisations and are strongly recommended [132].

It is also important to monitor glucose levels and to treat worsening hyperglycaemia in patients with COVID-19. According to a Chinese retrospective study, fasting glucose levels ≥126 mg/dL at admission were an independent predictor of increased mortality in patients with SARS-CoV-2 infection and without diabetes [133]. A retrospective exploratory study using continuous glucose monitoring has shown that patients with diabetes and COVID-19 have an increased risk of adverse outcomes and prolonged hospitalisation with glucose levels >160 mg/dL and <70 mg/dL and a high coefficient of variation [134]. Regarding diabetic patients infected with SARS-CoV-2 who are at risk of metabolic decompensation in the form diabetic ketoacidosis or a hyperosmolar hyperglycaemic state, clinicians should take notice of the tight control of diabetes [116]. The therapeutic aims in terms of plasma glucose concentrations during the infection are 4–8 mmol/L (72–144 mg/dL) in patients under ambulatory control and 4–10 mmol/L (72–180 mg/dL) in hospitalised patients [101].

During the mild course of COVID-19, it is necessary to continue the anti-diabetic treatment prescribed before the infection with one exception—SGLT2is, which may potentially cause dehydration or lead to euglycaemic diabetic ketoacidosis [135], but some authors allow their use with caution in a mild ambulatory course [3]. It is possible to follow treatment based on sulfonylurea but careful adjustment of the patient’s actual food intake and risk of hypoglycaemia should be performed [117].

**Figure 2:**
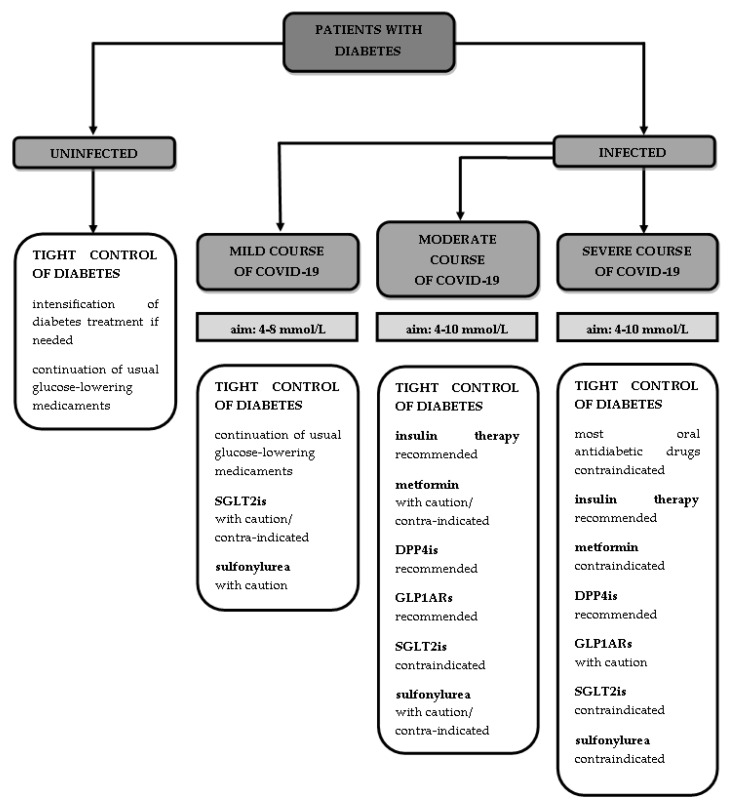
Recommendations pertaining to diabetes treatment during COVID-19 pandemic.

Regarding patients with a moderate or severe course of the infection, who should be hospitalised, treatment based on insulin seems to be a good option to maintain target glycaemia levels. Despite the fact that insulin therapy is correlated with a poor prognosis [63], it is still the main way to lower blood glucose levels in hospitalised patients, as most oral antidiabetic drugs are contraindicated [79,101] and tight diabetes control is required. It is not recommended to continue metformin treatment during the hospitalisation of patients with a moderate or severe course of COVID-19, because of the possibility of triggering lactic acidosis, especially under conditions of hypoxia [101]; however, some authors permit the use of metformin in hospitalised people with moderate disease with adequate caution, highlighting its anti-inflammatory properties [135]. The authors of a few guidelines propose continuing treatment based on DPP4 inhibitors, as there is no evidence of the need to discontinue the supply of this type of drug and they may be used safely in a broad spectrum of severity of the disease [101,135]. Similar recommendations have been made considering the use of GLP1 analogues [101,135]. During the hospitalisation of patients with moderate or severe COVID-19, SGLT2 inhibitors are strongly not recommended due to the risk of diabetic ketoacidosis, dehydration and acute kidney injury, as well as sulfonylurea because of the risk of severe hypoglycaemia, especially under in the presence of a loss of appetite or irregular food intake, which characterises the course of SARS-CoV-2 infection [101,135]. A short summary of the recommendations on diabetes treatment during the COVID-19 pandemic is presented in Figure 2.

## 5. Conclusions

COVID-19 is a new, not fully understood, disease which has affected a large number of people worldwide. The widespread occurrence of SARS-CoV-2 and its high infectiousness mean that the attention of scientists should be turned to better understanding the pathophysiology underlying COVID-19 and its impact on coexisting diseases. Antidiabetic drugs, used not only during asymptomatic or mild SARS-CoV-2 infection, but also within the severe course of the disease, can influence the pathophysiological mechanisms of COVID-19 and affect disease prognosis. Because of the high incidence of diabetes, it is of crucial importance to consider the relationship between SARS-CoV-2 infection and impaired glucose metabolism and to establish the most advantageous way to manage diabetes during the infection, which may prevent worse outcomes in these patients in the future.

## Figures and Tables

**Table 1:** A summary of antidiabetic drugs, their mechanisms of action, their possible influence on the course of COVID-19 and current reports regarding impact of these drugs on the course of SARS-CoV-2 infection in patients with diabetes.

Antidiabetic Drug	Mechanism of Drug Action	Possible Influence on the Course of COVID-19	Current Reports on Outcomes in SARS-CoV-2 Infection in Patients with Diabetes
DPP4is	- inhibition of DPP4, affecting the incretin axis [36] - influence on other properties of DPP4, including immune effects [35,37,38,39]; - reduction of pro-inflammatory marker levels [52,53] - affecting the interaction between DPP4 and caveolin-1 [55,56]	- prevention of coronaviruses from entering host cells [49,50] - anti-inflammatory effects [51]	- SIDIACO study—reduction in mortality, improvement in clinical outcomes, increase in hospital discharges [61] - Mirani et al.—reduced mortality [62] - Chen and Yang et al.—no influence on mortality and clinical outcomes in patients with moderate-severe course [63] - Fadini et al.—no association [64] - CORONADO study—no association between DPP4is and clinical outcomes [65] - Dalan et al.—increased risk of intensive care admission [66]
GLP1RAs	- influence on the incretin axis - interaction with NF-ĸB signalling pathway [70] - inhibition of the production and the release of cytokines [71,72,75] - reduction of the infiltration of immune cells in organs [36,73,74] - improved survival, vascular dysfunction, haemostatic indicators [76] - reduction of chronic inflammation [73,77,78] - increased expression of ACE2 [79,80] - cardiovascular prevention [73,87]	- systemic anti-inflammatory properties - increased susceptibility to the infection - anti-inflammatory and anti-fibrotic properties [84] - stimulation of the synthesis of surfactant [84,85] - protection of type II pneumocytes and prevention of ARDS [86]	Lack of data
SGLT2is	- inhibition of renal glucose reuptake in proximal renal tubes - increased expression of ACE2 [79] - increased production of angiotensin (1–7) [91] - reduction of cardiovascular and renal complications [92,97] - anti-inflammatory properties [92] - decreased production of tumour necrosis factor, IL-6, monocyte chemoattractant protein 1 [93,94] - decreased lactic acidosis, influencing the acid–base balance inside a cell [95]	- increased susceptibility to the infection [79] - anti-oxidative and anti-fibrotic properties [91] - prevention of ARDS development [91] - prevention of cell injury during the infection [95] - protection from the severe course of the disease [95]	- Bossi et al.—lack of influence in severe pneumonia [98] - Dalan et al.—lower risk of mechanical ventilation [66] - DARE-19 trial—currently being conducted [99]
Metformin	- anti-inflammatory properties [103] - anti-thrombotic features [104] - reduction in oxidative stress and inflammation in endothelium [107] - activation of AMPK or PI3K/Akt signalling pathway [108]	- prevention of cytokine storm or thromboembolic event development [103,104] - stops the virus from entering cells [108]	- CORONADO study—reduced rate of death only as an antidiabetic medication [17] - Hariyanto et al., Lukito et al., Luo et al.—decreased mortality [109,110,111] - Cheng and Xin et al.—decreased intensive care unit admission rate in a dose-dependent fashion [112] - Gao et al.—increased severity of infection, higher number of life-threatening complications [113] - Zangiabadian et al.—improvement in clinical outcomes [114] - Cheng and Liu et al.—promotion of acidosis, but not mortality [115]
Pioglitazone	- regulation of the transcription of genes connected with glucose and lipid metabolism [120] - anti-inflammatory activities [121,122,123]	- inhibition of RNA synthesis and replication of SARS-CoV-2 [124]	Lack of data
Insulin	- downregulation of ACE2 receptors [127] - optimisation of glycaemia control	- reduction of the risk of SARS-CoV-2 infection - positive influence on inflammation and coagulation during the infection [128]	- Cheng and Yang et al.—higher risk of poor prognosis [63] - Cheng and Xin et al.—increased invasive ventilation rate [112] - CORONADO study—no association with death [17]

## Data Availability

Not applicable.
